# Hospital mortality in acute coronary syndrome: differences related to gender and use of percutaneous coronary procedures

**DOI:** 10.1186/1472-6963-7-110

**Published:** 2007-07-13

**Authors:** María J Aguado-Romeo, Soledad Márquez-Calderón, María L Buzón-Barrera, the Medical Practice Variations Andalusian Group

**Affiliations:** 1 Andalusian Agency for Health Technology Assessment, Regional Health Ministry, Avenida de la Innovación, Seville, Spain

## Abstract

**Background:**

To identify differences among men and women with acute coronary syndrome in terms of in-hospital mortality, and to assess whether these differences are related to the use of percutaneous cardiovascular procedures.

**Methods:**

Observational study based on the Minimum Basic Data Set. This encompassed all episodes of emergency hospital admissions (46,007 cases, including 16,391 women and 29,616 men) with a main diagnosis of either myocardial infarction or unstable angina at 32 hospitals within the Andalusian Public Health System over a four-year period (2000–2003). The relationship between gender and mortality was examined for the population as a whole and for stratified groups depending on the type of procedures used (diagnostic coronary catheterisation and/or percutaneous transluminal coronary angioplasty). These combinations were then adjusted for age group, main diagnosis and co-morbidityharlson score).

**Results:**

During hospitalisation, mortality was 9.6% (4,401 cases out of 46,007), with 11.8% for women and 8.3% for men. There were more deaths among older patients with acute myocardial infarction and greater co-morbidity. Lower mortality was shown in patients undergoing diagnostic catheterisation and/or PTCA. After adjusting for age, diagnosis and co-morbidity, mortality affected women more than men in the overall population (OR 1.14, 95% CI: 1.06–1.22) and in the subgroup of patients where no procedure was performed (OR 1.16, 95% CI: 1.07–1.24). Gender was not an explanatory variable in the subgroups of patients who underwent some kind of procedure.

**Conclusion:**

Gender has not been associated to in-hospital mortality in patients who undergo some kind of percutaneous cardiovascular procedure. However, in the group of patients without either diagnostic catheterisation or angioplasty, mortality was higher in women than in men.

## Background

Many studies have been published on gender-related mortality and outcomes in acute coronary syndrome [1-3]. Most of these studies have shown a higher unadjusted mortality rate in women. For instance, Vaccarino [4], in one of his longest studies on this topic, stated that unadjusted prognosis was worse in women. However, differences in mortality decrease after adjustment for age, co-morbidity, treatments and procedures.

Several factors have been considered as possibly related to a high mortality rate for women, namely, the onset of acute coronary syndrome at a later age; an association with a higher number of cardiovascular risk factors and higher co-morbidity [5-8]; longer delay prior to receiving healthcare [9,10] and lesser efforts both in terms of diagnosis and therapy received by women [11-13].

However, the effect of gender on mortality has not yet been well defined and there are few data on how important gender is as an independent determinant of disease course in acute coronary syndrome patients. Also, little is known on whether the gender link to mortality depends on having undergone interventional procedures or not. It has, however, been proven that women do not undergo these procedures so frequently as their male counterparts [14,15]; although it is also difficult to discern to what extent this difference is accounted for by clinical and prognostic issues.

The aim of this paper is to assess whether gender is a predictive variable for disease course in patients with acute coronary syndrome, in terms of in-hospital mortality. The paper is also intended to assess whether this association depends on having undergone a percutaneous cardiovascular procedure, for diagnostic and/or therapeutic purposes.

## Methods

### Study scope and subjects

This study was conducted at the thirty-two public hospitals within the Andalusian Public Health System between 1st January 2000 and 31st December 2003. This comprised all hospitalisation events recorded in the Minimum Basic Data Set (MBDS: data base that records all discharges, and that must be completed by all public hospitals in Spain) that met the following selection criteria:

- Main diagnosis of acute myocardial infarction (AMI) or unstable angina (UA). The corresponding codes for these diagnoses are 410.01, 410.11, 410.21, 410.31, 410.41, 410.51, 410.61, 410.71, 410.81, 410.91, 411.1, 413.0, 413.1 and 413.9 in the ninth revision of the International Classification of Diseases (ICD-9).

- Patients must be recorded as alive on arrival at hospital and admitted urgently. This criterion is intended to avoid any errors in those cases where the main diagnosis of AMI or UA was coded for patients with a previous history of ischaemic heart disease and who had been admitted for some other non-emergency cause.

After this initial selection, discharges for transfer to another acute-care hospital (5,187 records) were omitted so as to avoid including the same hospital admission in the study twice, given that these cases were generally patients who were referred from hospitals without interventional procedure facilities to high level centres. All records where gender was coded as indeterminate (113 records) were also ruled out, leaving a total of 46,007 hospital admissions for acute coronary syndrome.

### Variables examined

In-hospital mortality was the dependent variable. The main independent variables were gender and performance of percutaneous procedures. All the procedure fields included in the MBDS were searched to identify all percutaneous procedures performed. The performance of any or none of the following procedures was identified among the selected admissions for acute coronary syndrome: diagnostic cardiac catheterisation (ICD-9 code: 37.21, 37.22, 37.23, 88.55, 88.56, 88.57) and percutaneous transluminal coronary angioplasty (PTCA) (ICD-9 code: 36.01, 36.02, 36.05, 36.09, 36.06, 36.07, 36.09). A three-category variable was then devised: performance of diagnostic catheterisation alone; performance of diagnostic catheterisation and PTCA; no procedure performed.

Other explanatory variables that may act as confounding or effect-modifying variables included:

- Age, in four age groups (<45 years, 45–64 years, 65–74 years, and >74 years).

- The main diagnosis recorded in the MBDS (UA or AMI).

- Co-morbidity studied by applying the Charlson score (ChS) (Table 1), constructed on diagnostic fields included in the Basic Minimum Data Set (MBDS). This score has been validated and proven to predict mortality at 30 days and one year post myocardial infarction [16,17]. This variable was stratified in four categories for further analysis: 1: (ChS = 0, control), 2: (ChS = 1), 3: (ChS = 2) and 4: (ChS ≥ 3). The ICHCALC 1.1 software was used to calculate ChS – this programme was run on Microsoft Access^©^.

**Table 1:** Weighting of Concurrent Diseases Included in the Charlson Score*

Acute myocardial infarction	1	Diabetes, chronic complications	2
Congestive heart failure	1	Hemiplegia or paraplegia	2
Peripheral cardiovascular disease	1	Renal disease	2
Dementia	1	Malignant tumours	2
Chronic pulmonary disease	1	Moderate/severe hepatic disease	3
Peptic ulcer	1	Solid metastatic tumour	6
Mild hepatic disease	1	AIDS	6
Mild-moderate diabetes	1		

*AIDS indicates acquired immune deficiency syndrome.

- A secondary diagnosis of diabetes mellitus in any hospital admission (coded ICD-9 code: 250.00 to 250.93). Given its relevance, this co-morbidity was examined independently.

### Ethical issues

This is an observational study (the diagnostic and therapeutic procedures were all performed in the normal course of care for patients with ACS and not in an experimental context). The MBDS data were lent by the Andalusian Regional Health Authority for this study. The data were sent to the authors of this paper once the clinical record number had been encrypted and the patients' names and surnames deleted. As a result, the use of all data complies with Spanish regulations on data protection.

### Statistical analysis

A descriptive analysis was conducted on in-hospital mortality, according to the different independent variables. The association between gender and mortality was analysed from a stratified standpoint according to the use made of percutaneous procedures (three subgroups: patients who had undergone no procedure at all, patients who only underwent diagnostic catheterisation and patients who underwent diagnostic catheterisation and a PTCA). The magnitude of the association for each group was evaluated by the odds ratio (OR), with a 95% confidence interval and the χ^2 ^test.

Multivariate logistic regression analysis by the forward stepwise method was also performed with in-hospital mortality as the dependent variable. One analysis was performed for the population as a whole, examining gender, use of procedures, age, main diagnosis and co-morbidity as the independent variables. A further analysis was conducted for each of the population subgroups defined according to the use of percutaneous procedures, using gender as the main independent variable and adjusting for the remaining variables.

The SPSS 12.0 statistical package for Windows was used for all analyses throughout the study.

## Results

### In-hospital mortality according to demographic and clinical features

During hospitalisation, mortality was 9.6% (4,401 cases out of 46,007), with 11.8% for women and 8.3% for men. (Table 2) Mortality was higher among older patients (16% of patients over 74 years) and among patients diagnosed with AMI. Also, mortality rises with increased co-morbidity – as measured by the Charlson score (Table 2).

**Table 2:** In-hospital mortality according to demographic and clinical features.

		**LIVE DISCHARGE**		**DEATH**		**p**
		Total N	%	Total N	%	
		41,606	90.4	4,401	9.6	
**Gender**						<0.0001
	**Male**	27,148	91.7	2,468	8.3	
	**Female**	14,458	88.2	1,933	11.8	
**Age**						<0.0001
	**<45 years**	1,865	97.5	47	2.5	
	**45–64 years**	12,822	96.0	528	3.9	
	**65–74 years**	13,817	91.2	1,330	8.8	
	**>74 years**	13,102	84.0	2,996	16.0	
**Diagnosis**						<0.0001
	**UA**	18,859	97.5	493	2.5	
	**AMI**	22,747	85.3	3,908	14.9	
**Co-morbidity**						<0.0001
	**ChS 0**	22,217	92.2	1,867	7.8	
	**ChS 1**	13,991	89.7	1,613	10.3	
	**ChS**	3,586	87.2	526	12.8	
	**ChS > 2**	1,812	82.1	395	17.9	

UA: Unstable angina. AMI: acute myocardial infarction. ChS: Charlson score.

Mortality was similar in both male and female patients in the 45- to 64-year (3.8% for women, 4.0% for men, p = 0.63) and over 74-year (16% in women, 15.4% in men, p = 0.37) age groups. There was a small, though statistically significant, difference in the 65- to 74-year group, (women 9.4%, men 8.4%, p = 0.047) and mortality was clearly higher among women in the under 44 year-old group (5% in women, 2% in men, p = 0.003).

The percentage of female diabetics who died was higher than the percentage of men who died with the same diagnosis (12.3% and 10.2% respectively).

### In-hospital mortality according to gender and use of procedures

No percutaneous procedure was performed in 80% of hospital admissions (86% for women, and 77% for men). In the remaining admissions, diagnostic catheterisation was performed in 2,264 women (13.8%) and in 6,926 men (23.4%), as well as PTCA which was performed in 1,150 women (7.0%) and in 4,254 men (14.4%).

386 patients underwent CABG (64 women and 332 men). Most of these patients had already undergone diagnostic catheterisation. Given the small number of CABG patients (0.8% of all patients in the study) and the low number of deaths recorded (79 deaths, i.e. 1.7% of all deaths), it was decided these patients should not be excluded from the analysis. The sample size for this subgroup was too small to allow for separate analysis.

In the stratification analysis according to use of procedures (without adjustment for other variables), greater mortality rates were seen among women than among men, both in the group without either diagnostic catheterisation or PTCA and in the group undergoing both procedures. No statistically significant differences were seen in mortality rates between men and women among patients where only diagnostic catheterisation had been performed (Table 3).

**Table 3:** In-hospital mortality by gender: Stratified analysis according to performance of percutaneous procedures.

		**LIVE DISCHARGE**		**DEATH**		
		Total N	%	Total N	%	p
		41,606	90.4	4,401	9.6	
**No procedure**						<0.0001
	**Male**	20,531	90.5	2,159	9.5	
	**Female**	12,320	87.2	1,807	12.8	
**Only diagnostic catheterisation**						0.377
	**Male**	2,521	94.3	151	5.7	
	**Female**	1,059	95.1	55	4.9	
**Diagnostic catheterisation and PTCA**						<0.0001
	**Male**	4,096	96.3	158	3.7	
	**Female**	1,079	93.8	71	6.2	

PTCA: percutaneous transluminal coronary angioplasty

In the multivariate analysis for the population as a whole (Table 4), in-hospital mortality was higher in women, in the older age group of patients, in patients with a diagnosis of AMI and higher co-morbidity. Lower mortality was seen in patients who underwent diagnostic catheterisation (performed alone or in association with PTCA) compared with patients who underwent no procedure at all. The variable diabetes did not enter in this multivariate model.

**Table 4:** Multivariate logistic regression models for the population as a whole: Variables associated with in-hospital mortality.

Variables (*)		**OR**	**95% CI**	
Gender				
	Female	1.14	1.06	1.22
Age				
	65–74 years	0.61	0.56	0.65
	45–64 years	0.28	0.25	0.31
	< 45 years	0.16	0.12	0.21
Diagnosis				
	AMI	7.27	6.60	8.01
Co morbidity (Charlson Score)				
	ChS 1	1.17	1.09	1.26
	ChS 2	0.45	0.13	0.62
	ChS > 2	2.30	2.03	2.62
Performance of procedures				
	Diagnostic Catheterisation	0.65	0.56	0.76
	Diagnostic Catheterisation and PTCA	0.42	0.37	0.49

* The reference categories were: male gender, age > 74 years, diagnosis of unstable angina, Charlson = 0, no procedure performed.

AMI: Acute myocardial infarction. ChS: Charlson Score. PTCA: percutaneous transluminal coronary angioplasty

However, this excess mortality seen in women when looking at the population as a whole is not present in all the subgroups defined according to the use made of procedures (no procedure, diagnostic catheterisation, diagnostic catheterisation and PTCA). As a result, in the multivariate analysis for each of these subgroups individually, gender has no impact in the two groups who underwent some kind of procedure, either for diagnostic or therapeutic purposes (Table 5). After adjusting for age, diagnosis and co-morbidity, the higher mortality rate for women was only seen in the subpopulation with no percutaneous procedure. In the three regression models, the remaining variables (age, diagnosis and co-morbidity) yield the same results as for the model including the population as a whole (Table 5).

**Table 5:** Multivariate logistic regression models for each population subgroup, defined according to use of percutaneous cardiovascular procedures: Variables associated with in-hospital mortality.

Variables (*)			**OR**	**95% CI**	
**No procedure (N = 36,817)**					
	Gender				
		Female	1.16	1.07	1.24
	Age				
		65–74 years	1.01	0.69	1.47
		45–64 years	0.28	0.25	0.31
		<45 years	0.17	0.12	0.24
	Diagnosis				
		AMI	7.53	6.80	8.33
	Charlson				
		ChS 1	1.17	1.08	1.27
		ChS 2	1.46	1.30	1.64
		ChS > 2	2.31	2.02	2.64

**Diagnostic Catheterisation (N = 3,786)**					
	Age				
		65–74 years	1.01	0.69	1.47
		45–64 years	0.43	0.28	0.66
		<45 years	0.08	0.02	0.35
	Diagnosis				
		AMI	4.17	2.89	6.00
	Charlson				
		ChS 1	1.22	0.89	1.68
		ChS 2	1.63	1.00	2.65
		ChS > 2	2.05	1.08	3.86

**Diagnostic Catheterisation and PTCA (N = 5,404)**					
	Age				
		65–74 years	0.64	0.45	0.92
		45–64 years	0.27	0.19	0.40
		<45 years	0.18	0.09	0.38
	Diagnosis				
		AMI	7.63	4.02	14.48
	Charlson				
		ChS 1	1.13	0.84	1.52
		ChS 2	1.02	0.56	1.86
		ChS > 2	2.73	1.42	5.25

* The reference categories were: male gender, age > 74 years, diagnosis of unstable angina, Charlson = 0.

AMI: Acute myocardial infarction. ChS: Charlson Score. PTCA: percutaneous transluminal coronary angioplasty

## Discussion and conclusion

No differences in terms of in-hospital mortality were seen in this study between women and men admitted urgently to hospital for UA or AMI who underwent diagnostic catheterisation and/or PTCA. However, higher mortality was seen among women in the group of patients who underwent no procedure at all.

A well-defined population was selected for this study, namely patients with a confirmed diagnosis leading to emergency admission, so as to ensure relatively homogeneous groups of populations of both men and women in terms of indication for interventional procedures. With a confirmed diagnosis in all cases as the cause for hospital admission, the hypothesis that the diagnosis of ACS is more difficult in women can be ruled out as the main underlying explanation for the differences found in the use of these procedures.

The main limitations in this study stem from the use of a secondary data source (MBDS). This does not allow for more in-depth analysis of the possible causes of the gender-derived differences, and explanations must be found based only on the variables included in the database (gender, age, procedures, co-morbidity and main diagnosis). As a result, the role of previous heart disease in these patients and other clinical variables, such as number of vessels involved, ejection fraction, shock upon admission, and treatment delivered, cannot be explored. Diagnosis of AMI has changed since the introduction of troponin in 2000, which may have had an impact on the results. Nonetheless, it might not affect the comparison between men and women. No distinction can be made between co-morbidities present at the time of admission and complications occurring during hospital stay or cause of death. Under-reporting of procedures in the MBDS cannot be ruled out. However, we do not think that such under-reporting will affect the comparison between men and women, i.e. the main aim of this paper.

Adjustment of in-hospital mortality has been widely used to assess the quality of healthcare provided for heart-disease patients [18]. However, there is some controversy over the differences in mortality rates in ACS among men and women, according to whether these patients undergo invasive cardiovascular procedures or not. Some studies have shown that women who undergo an invasive procedure for either diagnostic or therapeutic purposes die more often than men, especially if they are older and diagnosed with AMI. This has led to a less frequent indication for these procedures among women [4,12,13,15,19]. Other authors however, such as Vacek and Mehilli [20,21], detect no such differences.

The in-hospital mortality rates found in this study are similar to those reported in other studies also using the MBDS [22]. The results for the population as a whole show that gender is related to mortality; however, as other publications report [23], there is no difference in mortality between men and women when the analysis is limited to the group of patients who undergo some kind of percutaneous procedure.

As seen by other authors, age, a diagnosis of AMI and the patient's clinical status when undergoing interventional procedures show a stronger link with mortality than gender [21-27]. Several authors have suggested that older age, worse clinical status on admission, greater frequency of associated diabetes, together with a faster, more lethal course of AMI, may account for a higher in-hospital mortality rate among women [6,23,28-32]. However, a delay in diagnosis due to inadequate symptom identification, and subsequent delay in both drug and interventional therapy, may also have an impact [9,13,26]. Delay in delivering therapeutic measures in women is one of the determining factors in their disease course and is also a factor that could easily be changed [33]. All these factors, together with a lesser effort in diagnosis and therapy (i.e. admission to intensive care units, performance of diagnostic catheterisation and PTCA, etc.) may jointly account for the differences seen in mortality rates between men and women.

In this respect, this study revealed that percutaneous coronary procedures are associated with lower mortality, both when diagnostic catheterisation is performed alone (the results of this test may lead to positive medical decisions for the patient) and when followed by PTCA. The high percentage of patients undergoing neither of these procedures is striking, both among men and women, although this is even more remarkable among women. At least, this was the case in the context of this study (acute hospital admissions). Other variables, apart from gender, that were associated with diagnostic catheterisation and PTCA were age, diagnosis, and the Charlson score (wider use of this procedures in patients under 65, with a diagnosis of myocardial infarction and with higher co-morbidity); as has been reported elsewhere [12]. So, the observed association between cardiac catheterisation and reduced mortality may be partly related to lower baseline illness severity among patients referred for catheterisation.

However, the fact that a higher mortality rate for women (after adjusting for age, co-morbidity and diagnosis) is seen only in the subgroup of patients where no intervention at all was performed, suggests that there is still room for improvement in terms of providing percutaneous procedures for women.

The risk of death is determined more by variables such as age, diagnosis and the patient's clinical status than by the gender variable. However, gender may indeed be influencing current decision-making for more or fewer interventional measures, given that women comprise the group of patients where fewer diagnostic and therapeutic efforts have been made to date. This lesser effort may partly account for the higher mortality rates seen among female patients.

The gaps in our understanding of acute coronary syndrome and its behaviour in men and women must now be bridged. A different interventional attitude towards the diagnosis and treatment of female acute coronary syndrome patients may well contribute to enhanced disease control and improved mortality for this group of patients. Only by having valid studies will we be able to set optimum protocols of care and enhance survival outcomes.

## Competing interests

The author(s) declare that they have no competing interests.

## Authors' contributions

MJAR and SMC contributed towards study design and concepts, data acquisition, interpretation of data and manuscript preparation.

MLBB carried out the database preparation, and statistical analysis.

The remaining authors from the Medical Practice Variations Andalusian Group contributed towards discussion of study design and revised the manuscript.

All authors have approved the final version of the manuscript.

## Pre-publication history

The pre-publication history for this paper can be accessed here:


